# Investigating the Feasibility of Rapid MRI for Image-Guided Motion Management in Lung Cancer Radiotherapy

**DOI:** 10.1155/2014/485067

**Published:** 2014-01-12

**Authors:** Amit Sawant, Paul Keall, Kim Butts Pauly, Marcus Alley, Shreyas Vasanawala, Billy W. Loo Jr., Jacob Hinkle, Sarang Joshi

**Affiliations:** ^1^University of Texas, Southwestern Medical Center, Dallas, TX 75235, USA; ^2^University of Sydney, Sydney, NSW 2006, Australia; ^3^Stanford University, Stanford, CA 95305, USA; ^4^University of Utah, Salt Lake City, UT 84112, USA

## Abstract

Cycle-to-cycle variations in respiratory motion can cause significant geometric and dosimetric errors in the administration of lung cancer radiation therapy. A common limitation of the current strategies for motion management is that they assume a constant, reproducible respiratory cycle. In this work, we investigate the feasibility of using rapid MRI for providing long-term imaging of the thorax in order to better capture cycle-to-cycle variations. Two nonsmall-cell lung cancer patients were imaged (free-breathing, no extrinsic contrast, and 1.5 T scanner). A balanced steady-state-free-precession (b-SSFP) sequence was used to acquire cine-2D and cine-3D (4D) images. In the case of Patient 1 (right midlobe lesion, ~40 mm diameter), tumor motion was well correlated with diaphragmatic motion. In the case of Patient 2, (left upper-lobe lesion, ~60 mm diameter), tumor motion was poorly correlated with diaphragmatic motion. Furthermore, the motion of the tumor centroid was poorly correlated with the motion of individual points on the tumor boundary, indicating significant rotation and/or deformation. These studies indicate that image quality and acquisition speed of cine-2D MRI were adequate for motion monitoring. However, significant improvements are required to achieve comparable speeds for truly 4D MRI. Despite several challenges, rapid MRI offers a feasible and attractive tool for noninvasive, long-term motion monitoring.

## 1. Introduction

Respiratory motion causes significant uncertainties in tumor delineation, radiotherapy (RT) dose calculations, and delivery, particularly in the case of thoracic tumors (e.g., lung, liver) [1]. The management of respiratory motion has been an active area of research over the last decade. Several investigational as well as clinically implemented respiratory motion management strategies have been described in the literature [1]. However, a common limitation of most of these strategies is that they rely on image-guidance techniques that make simplifying assumptions about respiratory motion and do not adequately capture cycle-to-cycle variations which invariably occur in all patients. Modern motion-managed radiotherapy typically uses four-dimensional computed tomography (4DCT) as the tool of choice for pretreatment anatomic imaging (also termed as “CT simulation” or “CT-sim” in the literature). In this technique, CT projections are acquired over several respiratory cycles from successive “slabs” in the body. At the same time, an external surrogate (e.g., an optical marker) records the amplitude of respiration. Based on the surrogate motion trace, the reconstructed slices are sorted into 6–10 volumes over a single respiratory average cycle, where each volume represents a specific phase of respiration (inhalation through exhalation) [2–4]. This retrospectively reconstructed “movie” of a single respiratory cycle serves as the anatomical ground truth for all subsequent stages of radiotherapy (contouring, treatment planning, and dose delivery).

It is well recognized, however, that respiratory motion is far more complex than can be characterized by a single average cycle. Cycle-to-cycle variations such as baseline shifts and changes in the amplitude and/or frequency of the respiratory waveform are inadequately accounted for in 4DCT-based planning and can lead to significant geometric and therefore dosimetric errors [5]. Furthermore, binning CT projection data acquired over several cycles into a single cycle leads to severe image artifacts. For example, Yamamoto et al. found that 45 of 50 patients had at least one artifact, with mean magnitude of 11.6 mm (range: 4.4–56.0 mm) [6]. In a separate study, Persson et al. found that 4DCT artifacts caused significant uncertainties in the delineation of the gross tumor volume (GTV) in 16 out of 19 patients [7]. Finally, the equivalent dose for 4DCT is quite high (29–40 mSv), about 4 times higher than that for 3DCT (3–10 mSv) [8]. Such high imaging dose discourages long-term monitoring and frequent imaging. Due to these limitations, 4DCT-based image guidance provides an incomplete picture of respiration-induced spatial and temporal changes in the thoracic anatomy.

The aim of this work is to investigate the feasibility of using rapid magnetic resonance imaging (MRI) as a nonionizing imaging modality to capture long-term and/or frequent information about respiratory motion and its effects on the movement and deformation of lung tumors and surrounding critical organs. The fundamental difference and, therefore, advantage of cine MRI are that, unlike 4DCT, the MR image (i.e., slice or volume) is acquired prospectively, thereby capturing an actual instance of the patient anatomy, which is closer to reality compared to an average estimate of the anatomical state that is represented by 4DCT. Prospective acquisition also enables MRI to overcome the two main challenges that limit the utility of 4DCT images, namely, the ability to capture cycle-to-cycle variations and elimination of binning-related image artifacts. In addition, due to the fact that MRI does not involve ionizing radiation, there is no dose penalty for repeated imaging (as opposed to 4DCT).

The use of rapid cine-2D as well as 4D MRI for radiotherapy guidance has been previously reported in the literature. In cine-2D MRI, a slice of the anatomy is selected, at arbitrary orientation, and imaged repeatedly in time. 4D MRI is conceptually similar, except that in this case an entire volume is selected and imaged. Plathow et al. have reported cine-2D imaging of lung cancer patients at ~3 frames per second (fps) [9] and 4D imaging of malignant pleural mesothelioma patients at ~1 volume/s [10], under slow-breathing conditions using a 1.5 T scanner. Von Siebenthal et al. have reported on a 4D MR imaging technique using retrospective stacking of cine-2D slices [11]. Biederer et al. report 4D MRI of a ventilated chest phantom that uses porcine lung with embedded agarose nodules to simulate tumors [12]. More recently, Cai et al. have reported a 4D MRI study of a moving phantom using a technique that uses retrospective sorting of cine-2D slices [13]. To our knowledge, there has been no systematic study of rapid lung MRI in the context of image-guided radiotherapy (IGRT) motion management under realistic (prospective acquisition, free-breathing human subjects) conditions.

In this work, we present a pilot investigation of prospective rapid cine-2D and cine-3D (commonly termed as “4D” in radiotherapy and the MRI literature) MRI of two nonsmall-cell lung cancer (NSCLC) patients under free-breathing conditions, without externally administered contrast. Subsequently, we compute and analyze the motion trajectories of tumors and structures of interest. Our current goal is to demonstrate the feasibility and the utility of rapid MR imaging to monitor respiratory motion over multiple cycles and obtain guidance information about the motion, deformation, and the interplay between lung tumors and surrounding critical organs. Our long-term goal (beyond the current scope) is to use the information obtained from rapid MRI to augment and potentially correct 4DCT images.

## 2. Methods

### 2.1. Imaging of NSCLC Patients

Two NSCLC patients were imaged following informed consent. Patient number 1 was a 67-year old female with an ~40 mm diameter right midlobe tumor. Patient number 2 was an 80-year old male with an ~60 mm diameter left upper-lobe tumor. Both patients were scanned on a 1.5 T scanner (GE Signa). Both patients were scanned in the supine position, under free-breathing conditions and without externally administered contrast. For each patient, a 4-channel cardiac coil was centered around the tumor. cine-2D time series in the coronal and sagittal planes were acquired using a balanced steady-state free precession (b-SSFP) sequence and the images were reconstructed using the vendor's in-built software. In all cases except one (Patient number 1, coronal series), half-Fourier acquisition was used in order to achieve higher imaging speed. In the case of Patient number 2 an additional 3D+t (4D) scan of a tumor-inclusive coronal slab (8 slices, each 5 mm thick) was acquired using the b-SSFP sequence in the 3D mode and in conjunction with parallel imaging (acceleration = 4). The 4D images were reconstructed using the autocalibrating reconstruction for Cartesian imaging (ARC) algorithm [14].  Table 1 summarizes the image acquisition parameters for the cine-2D and the 4D acquisitions.

### 2.2. Motion Analysis

For each time series from Table 1, the motion trajectories of the tumor and structures of interest were determined as follows. A fluid-flow-based deformable image registration, previously validated for RT applications [15–17], was applied to each time series to compute deformation vector fields (DVFs) across the temporal dimension. In order to reduce errors and achieve high computation speed (i.e., fewer iterations), the registration was performed in two stages-rigid registration which accounted for gross translation and affine transformations of the tumor and organs, followed by deformable registration, which accounted mainly for tumor and organ deformation. For each time series, a reference image was selected (typically at mid-inhale) and ~15 points each on the tumor boundary and the diaphragm were manually selected. Subsequently, the motion trajectory of each pixel on a contour was determined from the DVFs. The validity of using diaphragmatic motion as a surrogate for tumor motion was examined by calculating the correlation between the average motion trajectory of the pixels comprising the diaphragm boundary with the average trajectory of the pixels comprising the tumor boundary. The presence of complex motion such as tumor rotation and/or deformation was tested by comparing the motion trajectory of the tumor centroid with those of the selected points on the tumor boundary.

## 3. Results and Discussion

**Figure 1:**
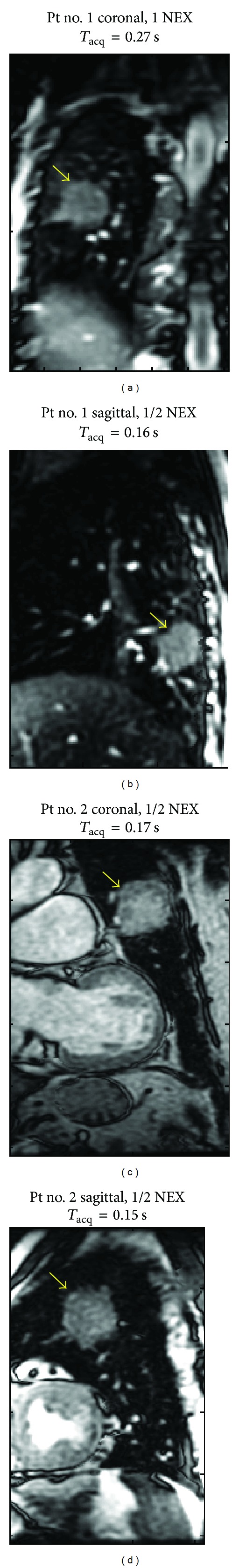
(a) Coronal and (b) sagittal real time MR images acquired from Patient number 1 with an ~40 mm diameter tumor (indicated by the arrows) in the right lower lobe. (c) Coronal and (d) sagittal real-time MR images from Patient number 2 with an ~60 mm diameter tumor in the left upper lobe.

**Figure 2:**
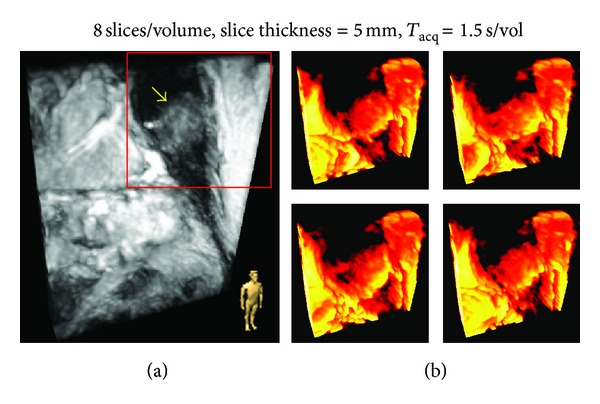
(a) bSSFP, 3D acquisition with parallel imaging (accn = 4) from Patient number 2. The arrow indicates tumor and the icon in the right bottom corner indicates the display orientation. (b) Surface-rendered volume of interest (red box in (a)) for four different respiratory phases.

Figure 1 shows MR images acquired from Patient number 1 (Figures 1(a) and 1(b)) and Patient number 2 (Figures 1(c) and 1(d)). The acquisition times per image ranged from ~0.15 to 0.27 s—speeds adequate for monitoring most respiratory motion. In each case, the tumor mass (indicated by an arrow) can be clearly delineated against the background of lung parenchyma.  Figure 2(a) shows a frame from the 4D acquisition from Patient number 2. A surface rendered tumor-inclusive volume-of-interest in four different respiratory phases is shown in Figure 2(b). Both the tumor as well as the surrounding anatomy exhibit significant deformation from phase to phase.

**Figure 3:**
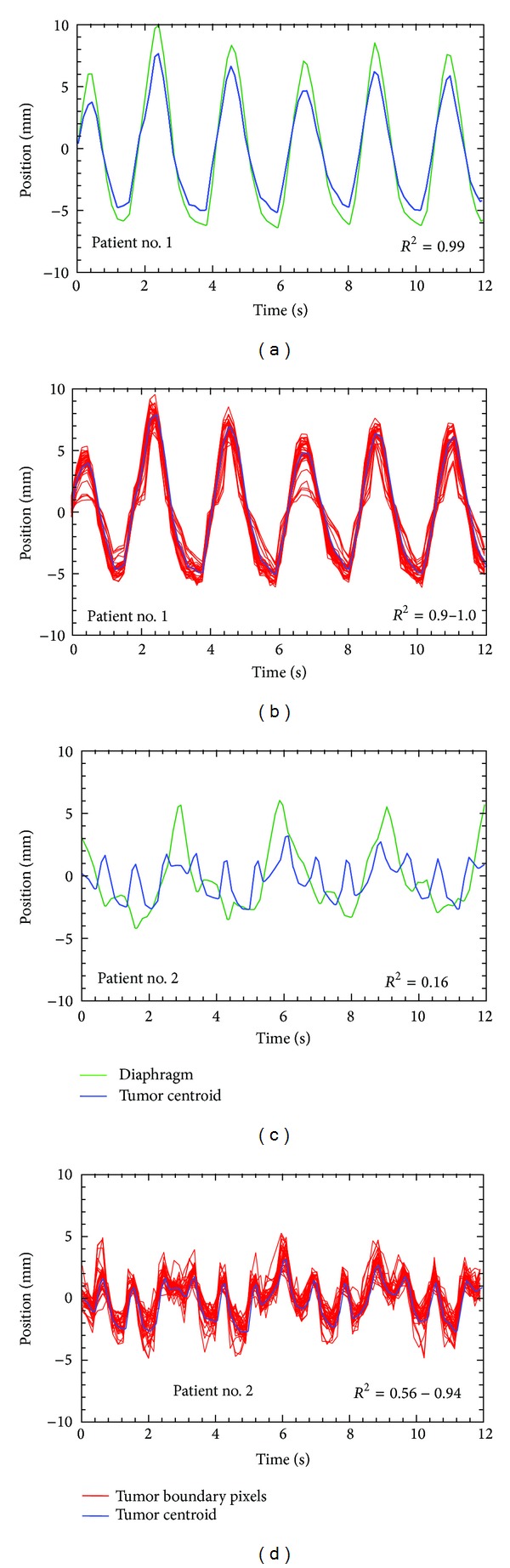
Motion trajectories of extracted from sagittal MRI time series from Patients number 1 and number 2 (Figures 1(b) and 1(d), resp.). ((a) and (c)) Mean-subtracted motion trajectories of the tumor centroid and the dome of the diaphragm for Patient number 1 and Patient number 2, respectively. ((b) and (d)) Trajectories of the tumor centroid and 15 points on the tumor boundary for Patient number 1 and Patient number 2, respectively.

Figure 3 shows motion trajectories extracted from two time series, one from each patient. MRI-based monitoring over multiple respiratory cycles yields some interesting observations. In the case of Patient number 1, there is little cycle-to-cycle variation in the respiratory pattern, as evidenced by the motion trajectory of the diaphragm. Furthermore, the motion of the tumor centroid is well correlated with the motion of the diaphragm (Figure 3(a); *R* ^2^ = 0.99) indicating that, in this case, diaphragmatic motion is an appropriate surrogate for tumor motion. Finally, the motion of individual points on the tumor boundary (i.e, pixels comprising the edges of the tumor mass) is well correlated with that of the tumor centroid (Figure 3(b); *R* ^2^ = 0.9 to 1.0), indicating the absence of any significant rotation or deformation in the tumor mass. In the case of Patient number 2, while the respiratory pattern is quite regular (as seen from the motion trajectory of the diaphragm), the motion of the tumor centroid is very poorly correlated with diaphragmatic motion (Figure 3(c); *R* ^2^ = 0.16) and shows significant cycle-to-cycle variation. This behavior indicates that, in this case, diaphragmatic motion is a poor surrogate for tumor motion. Furthermore, the motion of the tumor centroid is also relatively poorly correlated with that of individual points on the tumor boundary (Figure 3(d); *R* ^2^ = 0.56 to 0.94) indicating the occurrence of significant rotation/deformation of the tumor mass. The complex motion observed in Patient number 2 is likely due to the proximity of tumor to the cardiac wall, which almost touches the edge of the tumor (Figure 1(c)) and serves as a second actuator of motion (the first being the diaphragm). These results demonstrate that the current clinical practice of using the motion of the diaphragm (or external or internal surrogates for diaphragmatic motion) has significant limitations when the tumor mass is located in the proximity of other moving structures.

The goal of this work was to demonstrate the feasibility and the potential advantages of using rapid MRI as a pretreatment image-guidance tool for lung RT. These early results from rapid MRI of NSCLC patients show that, for guidance-quality imaging, the inherent contrast presented by the tumor mass and critical structures against the signal-poor lung parenchyma enables us to sacrifice SNR in order to achieve adequate acquisition speed to capture respiratory motion. Furthermore, in the case of Patient number 2, we observe that through long-term, prospective MR imaging, one can capture spatiotemporal effects that are not captured by 4DCT. This is due to the fact that 4DCT projections are sorted using an external surrogate for diaphragmatic motion, thereby implicitly assuming that a perfect correlation exists between diaphragmatic motion and tumor motion.

The choice of a 1.5 T scanner for this work was motivated by the fact that several lung motion investigations have been performed at this field strength [12, 18]. Observer studies comparing 1.5 T and 3 T scanners for lung MRI show that there is no significant difference in overall image quality [19, 20], suggesting that the expected benefits of higher SNR at 3 T are somewhat mitigated due to the accompanying increase in susceptibility artifacts. Furthermore, at this initial stage, we chose to use existing coils and sequences. As seen from the results, while this strategy was adequate for cine-2D imaging, very large improvements in acquisition speed are required for truly 4D MRI. This is evidenced by the fact that, even with the use of parallel acceleration = 4, the acquisition time for the 4D time series shown in Figure 2 was ~1.5 s/volume. Thus, there is much room for exploration of other rapid MRI sequences and for developing sequences specifically optimized for RT guidance. In particular, we expect the largest improvements in imaging speed to come from strategies based on sparse sampling and reconstruction such as k-t Broad-use Linear Acquisition Speed-up Technique (k-t BLAST) and its parallel imaging version, k-t SENSitivity Encoding (k-t SENSE).

Beyond the current scope, it is expected that the information obtained from rapid MRI (cine-2D or 4D) can be merged with that from 3DCT or 4DCT to create a fused pretreatment 4D image that combines the soft-tissue contrast and temporally dense information from MRI with the spatial accuracy and electron density information from CT. Admittedly, this is a nontrivial problem because one has to account for MRI artifacts, correct for geometric distortions of the anatomy due to the relatively narrow bore of the magnet, and develop robust multimodality image registration tools. Furthermore, since this was a feasibility study, the patients were not asked to lie in the treatment position for the MRI scan. However, for future studies which aim to fuse the MRI with CT, patients will be required to do so. However, if these challenges are addressed, fused 4D images would provide a more realistic picture of the behavior of thoracic anatomy over multiple respiratory cycles. Such guidance would enable the development of novel 4D treatment planning paradigms that explicitly account for effects such as baseline shifts and changes in abdominal versus thoracic breathing. Finally, several investigators are working on integrated MRI+linac designs [21–23]. Online prospective 4D MRI would enable such systems to perform real-time monitoring and, potentially, real-time beam adaptation.

## 4. Conclusion

We have investigated the feasibility of rapid MRI as a modality for image-based guidance in lung radiotherapy. While the acquisition speeds of cine-2D imaging are adequate for capturing most respiratory motion, significant further improvements are required to achieve comparable speeds for truly 4D MRI acquisition. Nevertheless, these early results indicate that rapid MRI offers a highly attractive, noninvasive imaging tool for respiratory motion management. The ability to perform dose-free, long-term monitoring over multiple respiratory cycles yields valuable information that is not currently available with 4DCT. We expect that such image-guidance will lay the groundwork for significantly better respiratory motion management in lung radiotherapy.

## Figures and Tables

**Table 1:** Summary of image acquisition parameters for rapid MRI of NSCLC patients.

	Image orientation	Acquisition (cine-2D/4D)	Voxel size (mm^3^)	FOV (mm^2^)	TE/TR (ms)	Flip angle (deg)	*N*_avg_	*T*_acq_ (s)
Patient 1	Coronal	cine-2D	2 × 3 × 5	240 × 240	1.70/3.41	50	1.0	0.273
	Sagittal	cine-2D	2 × 3 × 5	240 × 240	1.70/3.41	50	0.5	0.164

Patient 2	Coronal	cine-2D	2.4 × 3 × 5	240 × 240	1.68/3.16	50	0.5	0.165
	Sagittal	cine-2D	2.4 × 3.3 × 5	240 × 240	1.68/3.16	50	0.5	0.152
	Coronal (slab)	4D (//accn = 4)	2.4 × 3 × 5	240 × 240 (8 slices)	1.91/3.82	50	0.5	1.561
